# Preventing ototoxic hearing loss by inhibiting histone deacetylases

**DOI:** 10.1038/cddis.2015.252

**Published:** 2015-09-10

**Authors:** W S Layman, J Zuo

**Affiliations:** 1 Department of Developmental Neurobiology, St. Jude Children's Research Hospital, MS323, 262 Danny Thomas Place, Memphis, TN 38105, USA

The prevalence of hearing loss in the United States is currently estimated to affect one in five individuals over the age of 12 years.^1^ Most cases of hearing loss are of the sensorineural type, and are primarily caused by degeneration or damage to the mechanosensory hair cells in the inner ear. Unfortunately, humans and other mammals are unable to regenerate damaged hair cells, which results in permanent hearing loss. These mechanosensory hair cells have repeatedly been shown to be susceptible to ototoxicity from a multitude of drugs including aminoglycoside antibiotics, loop diuretics, platinum-based chemotherapy agents, and a number of non-steroidal anti-inflammatory drugs. Aminoglycoside antibiotics are among the most commonly used antibiotics worldwide in the treatment of serious Gram-negative bacterial infections. This is due to the low cost and high efficacy of aminoglycosides, despite the serious side effects of ototoxicity and nephrotoxicity that are associated with aminoglycoside treatment.

The formation of reactive oxygen species and activation of the c-Jun N-terminal kinase cascade are critical mediators of aminoglycoside-induced hair cell death (Figure 1).^2^ Aminoglycoside exposure also results in cytochrome *c* release from the mitochondria and caspase activation, which suggests that hair cells undergo caspase-mediated cell death.^2^ In addition, hair cells subjected to aminoglycoside antibiotics have increased histone deacetylation through recruitment of histone deacetylases (HDACs) to the chromatin.^3, 4^ Although HDAC inhibitors were shown to have a protective effect on neonatal hair cells subjected to aminoglycosides *in vitro*^3^ and *in vivo*,^4^ the precise mechanism underlying their protective effect in the inner ear was unknown.

Broad-spectrum and HDAC-specific inhibitors are known to have protective effects in a concentration-dependent manner in animal models of inflammation, neurodegeneration, and oxidative stress.^5, 6, 7^ However, HDAC inhibitor studies in the mammalian inner ear have been limited by their inability to cross the blood–labyrinth barrier and not cause ototoxicity. In our recent report published in *Cell Death Discovery*,^8^ we found that systemic delivery of the HDAC inhibitor, suberoylanilide hydroxamic acid (SAHA), both successfully crosses the blood–labyrinth barrier and does not adversely affect hearing thresholds in adult mice.^8^ Systemic SAHA treatment was found to offer almost complete protection against hair cell loss from acute ototoxic insult (kanamycin+furosemide).^8^ Mice receiving both ototoxic insult and SAHA were found to have little to no hair cell loss and normal hearing thresholds.^8^ Interestingly, SAHA treatment correlated with RelA K310 acetylation and RelA localization in the nucleus of hair cells from which it is normally absent following aminoglycoside treatment.^8^ Acetylated RelA K310 in the hair cell nuclei causes activation of the Nf-*κ*B pro-survival pathway leading to the expression of pro-survival genes such as *Cflar* (*cFLIP*) and *Bcl2l1* (*Bcl-xL*).^8^ Similar to other neuroprotection studies, the expression of pro-survival genes *Cdkn1a* (*p21*) and *Hspa1a* (*Hsp70*) were also increased in SAHA-treated mice, whereas the expression of the pro-apoptosis gene *Bcl2l11* (*Bim*) was significantly decreased.^8^

Another key finding from this study was that HDAC inhibition does not facilitate hair cell regeneration in adult mice that ectopically express the hair cell differentiation factor, *Atoh1*. Previous *in vivo* studies of hair cell regeneration had found that induction of ectopic *Atoh1* in supporting cells during the first postnatal week, leads to the formation of hair cell-like cells.^9^ However, *Atoh1* induction in adult mice cannot facilitate the formation of hair cell-like cells,^9^ which suggests that cochlear-supporting cells lose their cellular pliancy and capacity for cellular reprogramming during inner ear maturation. Although studies outside of the hearing field have shown that inhibitors of epigenetic events such as histone deacetylation are able to improve reprogramming efficiency,^10, 11^ HDAC inhibition in the inner ear combined with ectopic *Atoh1* expression was unable to facilitate cellular reprogramming.^8^ Taken together, these data indicate that hair cell lineage-specific genes in the mammalian supporting cells are tightly regulated by less dynamic epigenetics marks such as histone methylation and DNA methylation. Future reprogramming studies in the inner ear will need to focus on overcoming these more stable forms of epigenetic regulation.

Previous studies of aminoglycoside ototoxicity found that only outer hair cells in the organ of Corti misregulate the pro-survival Nf-*κ*B transcription factor complex (p50+RelA/p65).^12^ Our data indicate that Nf-*κ*B misregulation in hair cells in response to aminoglycoside antibiotic ototoxicity is caused by HDAC-mediated deacetylation of RelA/p65 at K310 resulting in RelA/p65 nuclear exclusion and degradation.^8^ RelA/p65 K310 deacetylation is directly mediated by HDAC3,^13^ whereas HDAC1 and HDAC2 also regulate RelA/p65 access to target genes (Figure 1). Interestingly, the Forkhead Box O transcription factor, Foxo3a, has also been shown to switch from upregulating the pro-apoptosis gene *Bcl2l11* (*Bim*) to upregulating the pro-survival gene *Cdkn1a/p21*^*CIP1*^ upon inhibition of HDAC2 (Figure 1).^14^ HDAC inhibitor treatment also blocks HDAC1-mediated deacetylation of Sp1 during oxidative stress leading to Sp1 hyperacetylation, which increases its DNA-binding affinity resulting in an upregulation of the pro-survival genes *Cdkn1a/p21*^*CIP1*^ and *Hspa1a/Hsp70* (Figure 1).^5, 15^ As HDACs regulate numerous cellular pathways in addition to those listed here, it is likely that the protective effect of SAHA is due to modulation of multiple signaling pathways. Regardless, our data provide evidence that inhibiting class I HDACs regulates the transcriptional activation of pro-survival pathways in response to ototoxic insult by regulating the acetylation status of transcription factors found at the crossroads of cell death and survival in the mammalian inner ear. Given that SAHA is already an FDA-approved drug, its use in clinical application to protect against ototoxicity may be the next step toward hearing protection in humans.

## Figures and Tables

**Figure 1:**
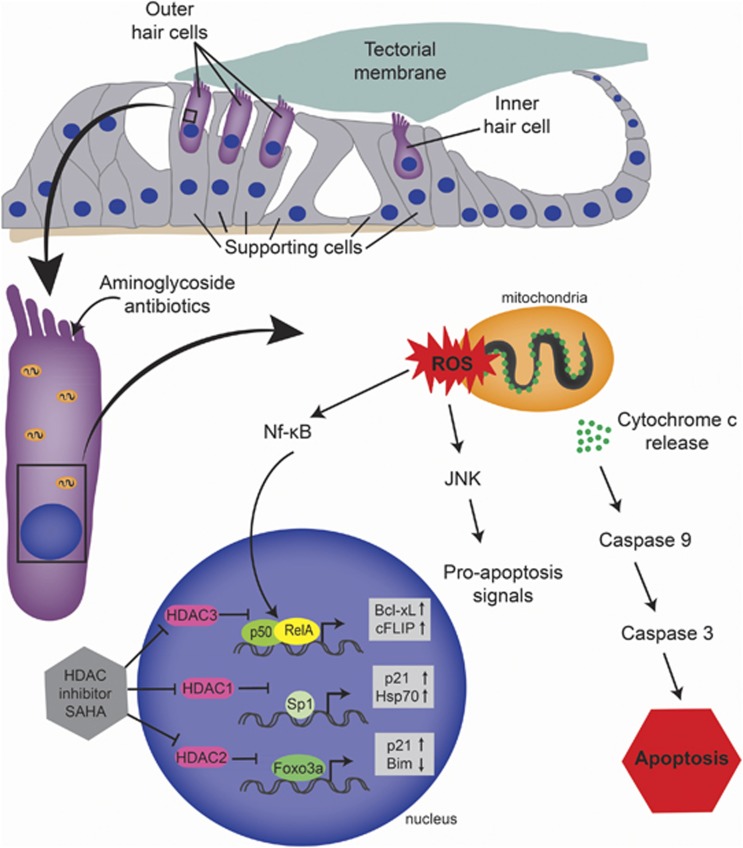
A simplified schematic diagram depicting aminoglycoside-induced hair cell death in the mammalian inner ear. Aminoglycoside exposure causes reactive oxygen species (ROS), stress kinases, and the caspase family of proteases to become activated in hair cells. Histone deacetylase (HDAC) inhibition using suberoylanilide hydroxamic acid (SAHA) stops the HDAC-mediated deacetylation of pro-survival transcription factors RelA (Nf-*κ*B), Sp1, and Foxo3a. Blocking the deacetylation of RelA K310 results in transcriptional activation of pro-survival genes *Cflar* (*cFLIP*) and *Bcl2l1* (*Bcl-xL*), which impedes aminoglycoside-induced apoptotic cell death. SAHA also inhibits Sp1 and Foxo3a deacetylation causing an increase in expression of pro-survival genes *Cdkn1a (p21*^*CIP1*^) and *Hspa1a (Hsp70)*. Abbreviations: c-Jun N-terminal kinase (JNK)

## References

[bib1] 1Lin FR et al. Arch Intern Med 2011; 171: 1851–1852. 22083573 10.1001/archinternmed.2011.506 PMC3564588

[bib2] 2Schacht J et al. Anat Rec (Hoboken) 2012; 295: 1837–1850. 23045231 10.1002/ar.22578 PMC3596108

[bib3] 3Chen FQ et al. J Neurochem 2009; 108: 1226–1236. 19141081 10.1111/j.1471-4159.2009.05871.x PMC3341988

[bib4] 4Wang J et al. Am J Otolaryngol 2015; 36: 242–248. 25554003 10.1016/j.amjoto.2014.11.003

[bib5] 5Ryu H et al. Proc Natl Acad Sci USA 2003; 100: 4281–4286.12640146

[bib6] 6Liu XS et al. Neuroscience 2012; 220: 313–321.22704966

[bib7] 7Robert C et al. Adv Cancer Res 2012; 116: 87–129. 23088869 10.1016/B978-0-12-394387-3.00003-3

[bib8] 8Layman WS et al. Cell Death Discovery 2015; 1: 1–7.

[bib9] 9Liu Z et al. J Neurosci 2012; 32: 6600–6610.22573682

[bib10] 10Huangfu D et al. Nat Biotechnol 2008; 26: 795–797.18568017

[bib11] 11Kim K et al. Nature 2010; 467: 285–290.20644535

[bib12] 12Jiang H et al. J Neurosci Res 2005; 79: 644–651.15672440

[bib13] 13Chen L et al. Science 2001; 293: 1653–1657.11533489

[bib14] 14Peng S et al. J Neurosci 2015; 35: 1250–1259.25609639

[bib15] 15Marinova Z et al. J Neurochem 2009; 111: 976–987.19765194

